# British Medicinal Plants

**Published:** 1890-12

**Authors:** Alfred Heath


### BRITISH MEDICINAL PLANTS.

ALFRED HEATH, M. D., F. L. S.

### NATURAL ORDER 2.—BERBERIDACEÆ.

*Berberis Vulgaris* (common Barberry, Pepperidge-bush, found in hedges and thickets). This very handsome shrub, with its pendulous racemes of golden yellow flowers (not forgetting its sharp three-parted thorns), is an object worthy of any garden or lawn on account of its showy appearance, both in its flowering state and when the yellow flowers are changed into bunches of scarlet berries, which are edible and often made into preserves. They are acid and astringent. The bark is used by dyers on account of its astringent properties and as an ingredient in a yellow dye. The common barberry has been used empirically with considerable success as a remedy in various disturbances of the liver, as a cooling drink in fever, in diarrhœa, in many kinds of disorders of the skin, for ague, bleeding piles, and as a tonic and astringent. It increases the appetite. It was also used as a remedy against the itch. The ashes of the burnt wood mixed with water, and the lye used as a wash for the hair are said to turn it yellow.

Jahr's *Homœopathic Materia Medica* gives a good proving of this drug. Amongst other things, it *produces* on the healthy body all the symptoms mentioned above, namely: Biliousness, nausea, and inclination to vomit, languor, various pains in the region of the liver and gall-bladder, and pressure, increased by external pressure, watery evacuations, flatulence, with burning, soreness, painful pressing, hemorrhoidal tumors, burning and itching, increased appetite almost to canine hunger, chills over the whole body with subsequent heat and increased thirst, heat of the hands and head, feeling of heat of the whole body, profuse night-sweats, sweats on the least exertion, lymphatic swellings,* general muscular languor and debility, feeling as if

* A case came under my care of a lady who had a swelling as large as a hen's egg in the left side of the chest over breast. It had been pronounced bruised, weakness almost to fainting when walking, pains excited or increased by movement. In the skin it produces burning, itching, pricking, as of mosquitoes, on the forehead, temples, cheeks, ears, lips, chin, scalp, and legs, obliging one to scratch, after which it disappears for a few minutes, or else reappears at a different place, leaving red spots after the rubbing; itching of the backs of the hands and fingers. The pains of Berberis more especially affect the left side.

*Berberis Aquifolium* (shining leaf, evergreen). This, although not an English plant, is common to our gardens and parks, and has lately been used in medicine. There is, I believe, no proving. Its properties are similar to the foregoing. It has been found especially efficacious in some forms of psoriasis, involving one-half of the face and nearly one-half of the body, down the sides and hips, also in scaly eruptions, like fish scales, on arms and legs, and in cases of general weakness and loss of appetite, despondency, etc.

### Natural Order 3.—Nymphæaceæ.

*Nymphœa Alba* (the white water-lily). Found in all parts of England, in rivers, lakes, and clear-water ditches. It was formerly used as a demulcent and anodyne. It was esteemed a powerful remedy for leucorrhœa, and in weakness left after venereal complaints; also for violent purgings and bloody stools.

*Nuphar lutea* (the yellow water-lily; brandy-bottle). Common in lakes and ditches in England. There is a smaller kind (*N. pumilum*) found in the Highland lakes. In some parts of Sweden the roots were, in times of scarcity, used as food, and are not unwholesome. This beautiful plant must be seen in all

---

by a surgeon to be encysted tumor, and operation recommended. Berberis in the 200th potency removed it in a week, but when first taken it produced one of its well-known symptoms, *most profound prostration.* Patient could not sit up all day, and was completely helpless. Such marked aggravation led me to give another dose at the end of fourteen days. I could not believe it was from the medicine. The prostration was again produced *as before*, and although the lady had no idea I had given her the Berberis, she said, "You have given me that medicine again."

its glory to be appreciated. It is a lovely sight to see hundreds of its large golden flowers dotted over the leaf-covered surface of a quiet pond. Quite a harvest is often reaped by sending the flowers to the London market (this also applies to the white water-lily). There is some proving of the plant mentioned in the *North American Journal of Homœopathy*, vol. III, p. 250; also in the *British Journal*, vol. XVI, p. 329. It is said to have cured some forms of leprous eruptions.

### Natural Order 4.—Papaveraceæ.

*Papaver Dubium* (one of the common red poppies). There is no proving of this plant; it has, however, been used in medicine in Germany.

*Papaver somniferum-Opium* (sleep-bearing poppy, white poppy, opium poppy). This is the plant from which opium is obtained. It is rarely wild in this country and is not considered to be indigenous, although it grows here perfectly, and at one time was cultivated to some extent, and the opium obtained from it was said to be equal, if not superior, to that obtained from Asia Minor, and as much as sixteen pounds of opium per acre being obtained; but the great reduction in price of foreign opium was fatal to it in a commercial point of view.

The mode of collecting the opium is by wounding the seed-vessel or poppy-head externally while yet green but of full size, which is done in the evening. The milky juice which flows freely from the incisions and dries and hardens in the air into a pale brown, adhesive substance, is collected in the morning by women and children, and forms opium (and from opium we obtain morphia, codeia, para-morphia, narcotine, and narceine and meconine), a drug that has done probably as much harm as good. The pernicious practice of taking or smoking opium has become the besetting sin of Turks, Chinese, and others. In countries where the prevailing religion forbids the use of alcohol, as in Turkey, it is in constant use as an indulgence, which if once commenced is rarely abandoned. The Turks call it "*afium*," and in the opium shops of Constantinople they take it in graduated doses from ten to fifty or a hundred grains a day. To make it palatable it is mixed with syrups or fruit juice; it is also made up in lozenges stamped with the words "*Mash Allah*," meaning "*work of God.*" It is also smoked. The Tartar couriers travel great distances with astonishing rapidity, and take little else to support them on their journeys. Its effects are to impair the digestive organs and to undermine the vigor of the whole body; it affects the mental energies, the memory fails, the victim becomes prematurely old and endures frightful sufferings after the effect of the dose subsides. As a drug opium is certainly of great value. In the old school it is mostly used as a sedative or to produce sleep—not natural but heavy, unrefreshing sleep from which the patient generally awakes unrelieved or worse than before, as it produces the most profound desire to sleep, but with total inability to do so. In Homœopathy this is an indication for its use (provided it has not been produced by taking opium or any of its alkaloids) and it will generally instantly relieve and cause refreshing natural sleep, in the most infinitesimal dose. In allopathy it is constantly used to produce a constipated state of the bowels in diarrhœa, and also in surgical operations. Constipation of the most obstinate nature, with headache, dizziness as if intoxicated, or with great heaviness of the head, drowsiness, and bewildered feeling is often entirely cured with a few small doses of opium. It also causes in healthy persons congestion of the brain, heaviness of the head, great desire to sleep, lethargy with snoring (one would almost think that the poppy flower, which always droops while in the bud, was an emblem of sleep, or the finger of Nature indicating its virtues or sphere of action ("Doctrine of Signatures"), eyes half closed, convulsed, lids hanging as if paralyzed. In natural congestion and inflammation of the brain, with great lethargy and eyes partly open (a common feature of this disease), with constipated bowels, etc., the effect of small doses of opium is really magical, its quickness of action must be seen to be believed. Unfortunately the abuse of one of God's greatest blessings to man is in the case of opium often one of the greatest evils.

